# Contemporary analysis of phrenic nerve injuries following cryoballoon-based pulmonary vein isolation: A single-centre experience with the systematic use of compound motor action potential monitoring

**DOI:** 10.1371/journal.pone.0235132

**Published:** 2020-06-25

**Authors:** Omar Anwar, Melanie A. Gunawardene, Jannis Dickow, Katharina Scherschel, Christiane Jungen, Paula Münkler, Christian Eickholt, Stephan Willems, Nele Gessler, Christian Meyer

**Affiliations:** 1 Department of Cardiology, Asklepios Clinic St. Georg, Faculty of Medicine, Semmelweis University Campus Hamburg, Hamburg, Germany; 2 Department of Cardiac Electrophysiology, Heart and Vascular Centre, University Hospital Hamburg Eppendorf, Hamburg, Germany; 3 DZHK (German Center for Cardiovascular Research), partner site Hamburg/Kiel/Luebeck, Berlin, Germany; 4 Division of Cardiology, cardiac Neuro- and Electrophysiology Research Consortium (cNEP), EVK Düsseldorf, Düsseldorf, Germany; 5 Cardiac Neuro- and Electrophysiology Research Consortium (cNEP), Institute for Neural and Sensory Physiology, Medical Faculty, Heinrich Heine University Düsseldorf, Düsseldorf, Germany; Klinikum Region Hannover GmbH, GERMANY

## Abstract

**Background:**

Phrenic nerve injury (PNI) remains one of the most frequent complications during cryoballoon-based pulmonary vein isolation (CB-PVI). Since its introduction in 2013, the use of compound motor action potential (CMAP) for the prevention of PNI during CB-PVI is increasing; however, systematic outcome data are sparse.

**Methods:**

The CMAP technique was applied in conjunction with abdominal palpation during pacing manoeuvres (10 mV, 2 ms) from the superior vena cava for 388 consecutive patients undergoing CB-PVI between January 2015 and May 2017 at our tertiary arrhythmia centre. Cryoablation was immediately terminated when CMAP amplitude was reduced by 30%.

**Results:**

Reductions in CMAP amplitude were observed in 16 (4%) of 388 patients during isolation of the right veins. Of these, 11 (69%) patients did not manifest a reduction in diaphragmatic excursions. The drop in CMAP amplitude was observed in 10 (63%) patients during ablation of the right superior pulmonary veins (PVs) and in 7 (44%) patients during ablation of the right inferior PVs. Postprocedural persistent PNI was observed in three of four patients for a duration of 6 months, with one of these patients remaining symptomatic at the 24-month follow-up. One of the four patients was lost to long-term follow-up.

**Conclusions:**

All PNIs occurred during right-sided CB-PVI and were preceded by a reduction in CMAP amplitude. Thus, the standardized use of CMAP surveillance during CB-PVI is easily applicable, reliable and compared with other studies, results in a lower number of PNIs.

## Introduction

Cryoballoon (CB)-based catheter ablation is increasingly being used for the treatment of atrial fibrillation (AF). Although the success rates of second-generation CB (CB-G2) catheter ablation are promising in terms of freedom from AF and a shorter procedure time, persistent phrenic nerve injury (PNI) has been reported in up to 7.1% of cases [1]. Although both the left and the right phrenic nerve can theoretically be injured, the right phrenic nerve is most susceptible to injury during cryoablation of the right pulmonary veins (PVs), due to their close proximity to one another [2,3].

Electromyography of the phrenic nerve is widely used as a diagnostic tool in neurological disorders that affect respiration [4,5]. Compound motor action potential (CMAP) is the recorded summation of muscle potential waveforms produced by healthy innervated muscle fibres and has been described in motor nerve conduction studies [4]. Although the use of diaphragmatic CMAP recordings during catheter ablation for prevention of PNI is gaining popularity in daily clinical routine, systematic data on the relevance of CMAP to predict PNI are still limited.

Here, we analyzed the usefulness of systematic application of this electromyographic monitoring technique for the prevention of PNI in 388 patients during CB-pulmonary vein isolation (PVI).

## Methods

### Demographic data

This single-centre, observational, non-randomized study was conducted on 388 consecutive patients with AF who underwent first-time CB-PVI between January 2015 and May 2017 at our tertiary arrhythmia centre. All patients provided written informed consent. The local institutional review board of the University of Hamburg approved this study. Exclusion criteria for the procedure included repeat procedures, hyperthyroidism, pregnancy, the presence of intracardiac thrombi and limited life expectancy (less than 1 year) [6].

### Catheter ablation procedure

The ablation procedure has previously been described in detail [6]. Transesophageal echocardiography was used to rule out thrombus formation in the left atrial appendage prior to the procedure. Ablation procedures were performed under deep sedation using a continuous infusion of propofol (1 mg/mL, B. Braun, Melsungen, Germany), as well as boluses of fentanyl (0.1 mg/mL, Rotexmedica, Trittau, Germany). Intraprocedural anticoagulation was initiated by a bolus injection of intravenous heparin (heparin sodium, 25000 IU/5 mL, Rotexmedica) to maintain activated clotting time above 300 s.

An octapolar diagnostic catheter (Inquiry^™^, 2-2-2 mm spacing, 6 French, St. Jude Inc., St Paul, MN, USA) was placed in the coronary sinus via a femoral approach. After a single trans-septal puncture (BRK-1^™^, St. Jude) via a SL0-Sheath (Fast-Cath, St Jude, USA), an angiography (Iomeprol, IMERONw 350 mg/mL, Bracco Imaging, Konstanz, Germany) of the left atrium was performed in order to assess its anatomy and to gain an overview of the pulmonary veins. Subsequently, the Sl-0 Sheath (Fast-Cath, St Jude, USA) was replaced with a 12 F steerable sheeth (FlexCath^™^). The 28-mm CB-G2 (Arctic Front Advance^™^, Medtronic Inc., Minneapolis, MN, USA) was then introduced into the left atrium via the FlexCath^™^. PV mapping to record electrograms was performed before, during and after freezing using an endoluminal spiral mapping catheter (Achieve^™^, Medtronic). To assess the exact position of the inflated balloon in relation to the PV ostium, contrast medium (Iomeprol, IMERONw 350 mg/mL, Bracco Imaging, Konstanz, Germany) was injected from the distal lumen of the CB-G2 with the aim of complete PV occlusion before freezing. Occlusion was categorized (1 to 4), with perfect circumferential occlusion of the PV defined as 4 (no lack of contrast medium) and imperfect occlusion as 1 (ineffective position with a massive flow of contrast medium). Primary target application time was 240 s. A single 180-s freeze technique was used when real-time isolation occurred within 60 s of ablation [7]. Bonus freezes were not applied. During ablation of the right PV, the phrenic nerve was paced from the superior vena cava (SVC) via the most distal electrode of the octapolar diagnostic catheter. In case phrenic nerve capture was insufficient or non-existant, a more proximal pole was utilized. In order to monitor esophageal temperature, a temperature probe was placed into the esophagus once the patient was sedated. Once the esophageal temperature approached 15°C, cryoablation was terminated with the double-stop technique [8].

### CMAP and CMAP monitoring

**Fig 1:**
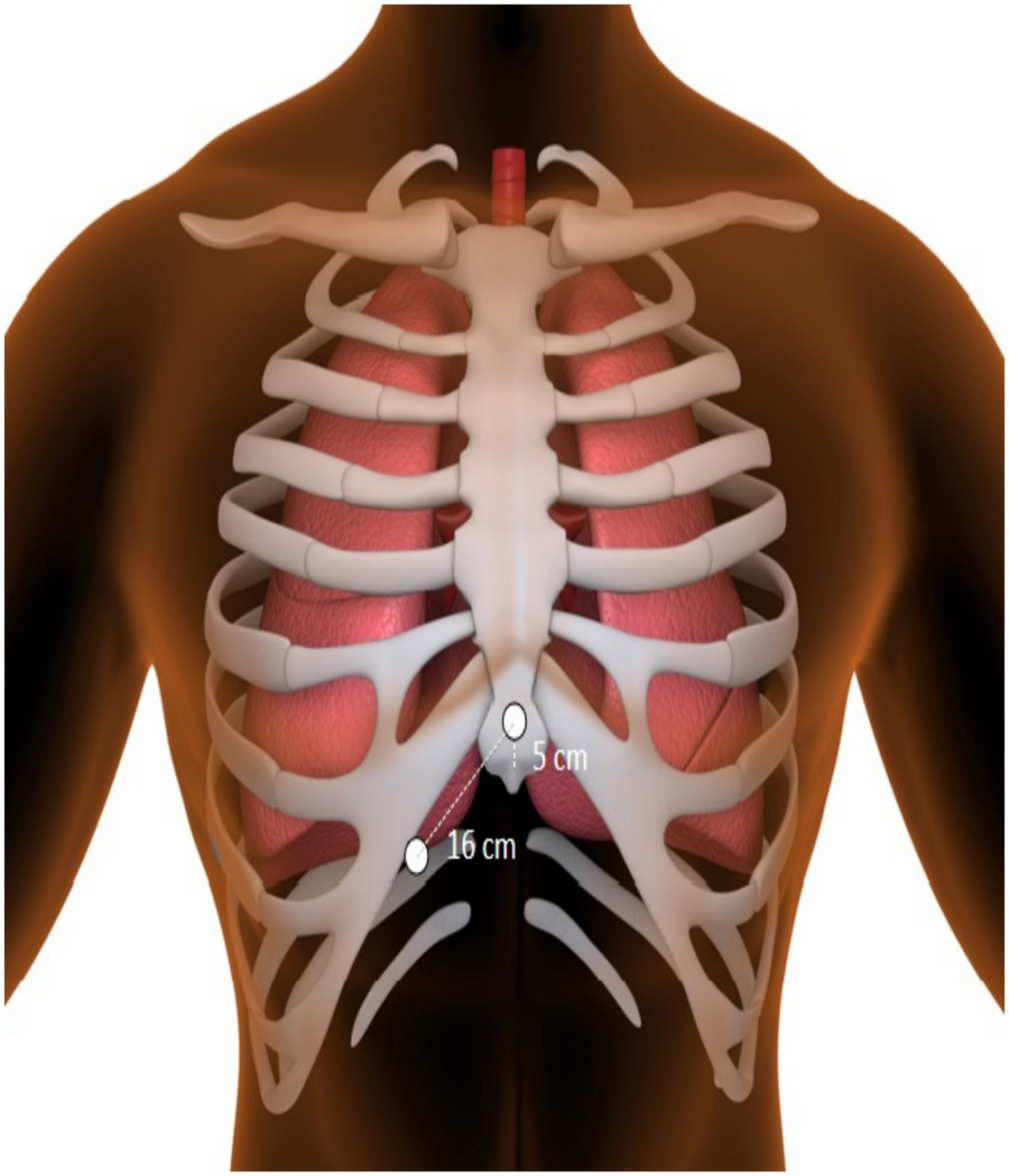
Placement of electrodes for CMAP.

**Fig 2:**
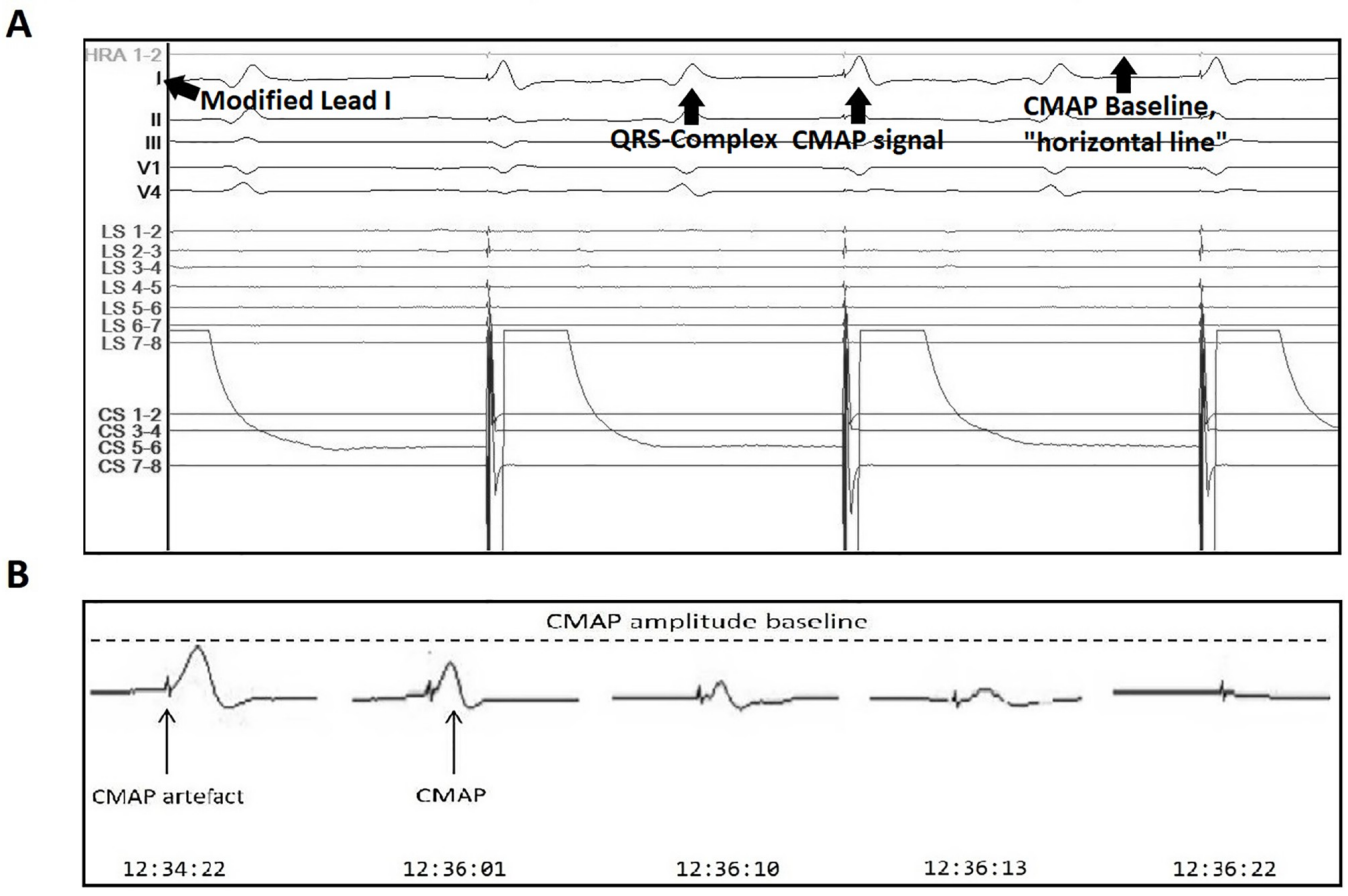
Phrenic nerve pacing. **A.** Phrenic nerve pacing through coronary sinus (CS) catheter via superior vena cava during cryoablation. Lead I is modified (see CMAP and CMAP monitoring) and the “horizontal line” is used as a visual aid in order to assess any changes in CMAP amplitude (baseline CMAP amplitude). **B.** Reduction in CMAP amplitude with complete loss of capture (left to right) during cryoablation of the RSPV. CMAP = compound motor action potential, RSPV = right superior PV.

CMAP monitoring, as previously described by Franceschi et al. [9], was recorded using two standard surface electrodes on the right hemithorax; the first was placed 5 cm above the xyphoid process and the second at a distance of 16 cm along the right costal margin [9] (see Fig 1). Diaphragmatic CMAP signals were analyzed in real-time by two experienced physicians and observed continuously throughout the procedure. As soon as a 30% reduction in CMAP amplitude or a decrease in diaphragmatic excursion was perceived, cryoablation was immediately terminated using the double stop technique [8]. In addition, the cryoballoon was pulled away from the pulmonary vein in order to increase the distance to the phrenic nerve [10]. The 30% reduction was visually assessed using a horizontal reference line placed above the modified lead I. An example of phrenic nerve pacing, reduction in the CMAP amplitude as well as use of the horizontal line is presented in Fig 2. If CMAP amplitude recovered, another freeze was attempted after placing the spiral mapping catheter in a different PV branch with a more antral position of the balloon, if possible [11]. If CMAP reduction subsequently occurred, the cryoablation was aborted again using the double-stop technique [8].

### Phrenic nerve injury and follow-up

We differentiated between transient and persistent PNI. We defined transient PNI as PNI with a manifest reduction in diaphragmatic excursions during the procedure that resolved toward the end of the procedure. Persistent PNI was defined as PNI with a manifest reduction in diaphragmatic excursions as well as radiological evidence of PNI that remained after the procedure, even if it resolved before discharge. For the purpose of comparing studies [2,7,12,13,14,15,16,17,18,19,20,21,22,23], incidences of PNI were re-categorized (if necessary) according to our definition. Once persistent PNI occurred, a chest X-ray was used to confirm diaphragmatic paralysis (using Hitzenberger’s test or the sniff test) [24] 24 h after the procedure. The affected patient was closely monitored for at least 48 h after the procedure. Additionally, patients were examined at a 3- and 6-month follow-up visit. During these, chest X-rays were repeated to determine the persistence of diaphragmatic paralysis. Regular physiotherapy, with breathing exercises focusing on phrenic nerve stimulation, was recommended to all patients [25].

### Statistical analysis

All data are presented as mean ± standard deviation for continuous variables and as numbers (%) for categorical variables. Sensitivity and specificity were calculated conventionally using a 2 × 2 contingency table. Data were collected and statistics were calculated using Microsoft Excel 2016 and WPS Spreadsheet 2017.

## Results

### Baseline and procedural characteristics

Baseline characteristics are summarized in Table 1. The mean age was 62.3 ± 10.6 years. Of the 388 patients, 245 were men (63%) and 143 were women (37%). The mean body mass index of these patients was 27 ± 4 kg/cm². A median 4-year history of AF was present prior to the procedure. Procedural baseline characteristics are summarized in Table 2. In total, 1528 PVs were targeted during cryoablation, with a median minimum temperature of −46 ± 7°C. 99.5% (1520/1528) of all PVs were successfully ablated. Ablation was not completed in 8 PVs due to capture loss of the phrenic nerve (n = 6) as well as due to a decrease in oesophageal temperature (n = 2). One right inferior PV (RIPV) (0.07%) was not ablated due to its small size. Expansion of the CB could have caused damage to the vessel itself. Tables 3 and 4 summarize the procedural data of cryoablation of the right-sided and left-sided PV, respectively.

**Table 1:** Baseline characteristics.

Number of Patients	388
Age (years)—mean ± SD	62.3 ± 10.6
Number of men (n, %)	245 (63)
Hypertension (n, %)	234 (60)
Diabetes (n, %)	31 (8)
Body mass index in kg/m²—mean ± SD	27 ± 4
Patients with PAF (n, %)	281 (72)
Patients with persistent AF (n, %)	107 (28)
Duration of AF in years—mean ± SD	4 ± 6

PAF = paroxysmal atrial fibrillation

**Table 2:** Procedural baseline characteristics.

CB-G2 28 mm (n, %)	388 (100)
Duration of procedure in minutes—mean ± SD	98 ± 29
Fluoroscopy time in minutes—mean ± SD	16 ± 7
Total number of cryoapplications per PV—mean ± SD	1.5 ± 1
Total cryoablation time per PV (seconds)—mean ± SD	310 ± 156
Average occlusion quality—mean ± SD	3.7 ± 0.5
Total number of PVs targeted for ablation	1528
Minimal temperature (°C)—mean ± SD	−46 ± 7
Capture loss of phrenic nerve (n)	6
Dislocation of CS catheter (n)	9

PV = pulmonary vein, CS = coronary sinus

**Table 3:** Procedural data and PNI for right-sided PVs.

Total number of right-sided PV targeted for ablation:	765			
CMAP Reduction with paralysis or injury—number of patients	5			
CMAP Reduction without paralysis or injury—number of patients	11			
	**Right superior PV (RSPV)**	**Right inferior PV (RIPV)**	**Right middle PV (RMPV)**	**Right common PV (RCPV)**
Total number of veins—(n, %)	383 (50)	382 (49.9)	2 (0.3)	5 (0.6)
Occlusion quality—mean ± SD	3.8 ± 0.5	3.5 ± 0.6	3.5 ± 0.7	3.8 ± 0.4
Cumulative cryoablation time (seconds)—mean ± SD	316 ± 159	289 ± 123	240 ± 0	297 ± 150
Minimum temperature (°C)—mean ± SD	−49 ± 7	−44 ± 7	−41 ± 23	−45 ± 9
>30% reduction in CMAP amplitude—number	10	7	0	0
Transient phrenic nerve injury–number	0	1	0	0
Clinical phrenic nerve injury—(n, %)	2 (0.5)	2 (0.3)	0	0
Persistence of symptomatic phrenic nerve paralysis at 6 months—number	1	0	0	0

PNI = phrenic nerve injury, PV = pulmonary vein

**Table 4:** Procedural data for left-sided PV.

	Left superior PV (LSPV)	Left inferior PV (LIPV)	Left common PV (LCPV)
Total number of veins—(n, %)	373 (48.7)	373 (48.7)	20 (2.6)
Occlusion quality—mean ± SD	3.9 ± 0.3	3.8 ± 0.4	3.6 ± 0.5
Cumulative cryoablation time (seconds)—mean ± SD	337 ± 187	286 ± 126	531 ± 241
Minimum temperature (°C)—mean ± SD	−48 ± 6	−43 ± 7	−48 ± 6

PV = pulmonary vein

### Reductions in CMAP amplitude and PNI

Reductions in CMAP amplitude were observed in 16 (4%) of 388 patients during right-sided (septal) PVI. Of these 16 patients, 11 (69%) did not manifest a reduction in diaphragmatic motion or PNI. Reductions in CMAP amplitude during cryoablation of the right superior pulmonary vein (RSPV) and the RIPV occurred in 10/16 (63%) patients and 7/16 (44%) cases, respectively (in one patient CMAP reduction occurred during ablation of both the RSPV and RIPV). In 1 (6%) of these 16 patients, a temporary decrease in diaphragmatic motion was perceived during cryoablation of the septal PV in addition to a reduction in CMAP amplitude >30%, which resolved spontaneously toward the end of the procedure. Four patients had a persistent loss of phrenic nerve capture indicating PNI at the end of the procedure. We did not observe a difference between the reduction in CMAP amplitude between the cases of persistent PNI and transient PNI. All cases of PNI were preceded by a reduction in CMAP amplitude. The use of CMAP during CB-PVI showed a sensitivity of 25% and a specificity of 100%. Although ablation was interrupted under observed CMAP reduction in all 16 cases, all veins were isolated at the end of the procedure.

### Follow-up

Four (1%) of 388 patients had persistent PNI after the procedure, as confirmed by a sniff test. A total of 1/388 patients had persistent PNI still detectable at a follow-up time of 24 months; the PNI in this patient caused breathlessness and reduced mobility in daily activities. In the remaining three patients, the first was asymptomatic at discharge and was lost to long-term follow-up. The second patient also remained asymptomatic. Phrenic nerve recovery in this patient was confirmed via chest X-ray using the sniff test [24] during the 6-month follow-up visit. The third patient had symptomatic PNI; however, the PNI had also resolved spontaneously within 6 months.

Of the 16 patients with CMAP reduction, 1 patient underwent a repeat AF ablation procedure. Interestingly, all veins required re-isolation except the RIPV, although the initial drop in CMAP amplitude occurred during ablation of the RIPV.

## Discussion

### Main findings

PNI still remains one of the most frequent complications during CB-PVI, although significant efforts have been made to develop more effective ablation techniques [26–30,14,22,23]. The main findings of this study are as follows: (1) Using CMAP results in a relatively low number of PNIs during CB-based PVI compared with other studies; (2) All cases of PNI in this study were preceded by a drop in CMAP amplitude; and (3) A drop in CMAP amplitude mainly occurs in the RSPV (63%, n = 10), though also in a relevant number of patients during ablation of the RIPV (44%, n = 7).

One of the challenges of utilizing CMAP reliably was to ensure the maintenance of a stable catheter position in the SVC during ablation of the septal veins as well as the avoidance of a loss of capture of the phrenic nerve. In 1.5% (n = 6) of the patients the CS catheter dislocated and in 2.3% of the patients (n = 9) a loss of phrenic nerve capture occurred. The stability and the presence of consistent phrenic nerve capture is very important, as a lack of good contact and loss of capture due to catheter manipulation may mimic PNI and may result in premature cessation of the procedure [27]. These elements also explain the low sensitivity calculated in this study. Factors influencing the CMAP signal, among which include breathing excursions, have been described in detail previously [31].

In our study, 3% of patients (n = 12) had a true decrease in CMAP amplitude that resulted in the immediate termination of cryoablation, thus avoiding imminent PNI. Our findings, including PNI in both the RSPV and RIPV using the CB-G2, is partially contrast with the current literature, which states that PNI occurs mostly during CB-PVI of the RSPV [3]. Our study demonstrates that the RIPV can also be susceptible to PNI, indicating that additional attention should be given during ablation of the RIPV.

#### CMAP vs. tactile feedback of diaphragmatic excursions

Despite the encouraging evidence that supports CB-based PVI as a valuable approach for the treatment of patients with drug-refractory symptomatic AF, PNI remains a relevant procedural challenge. Table 5 shows a series of studies over the course of the last few years, comparing PNI incidence between studies that used CMAP vs. tactile feedback of diaphragmatic excursions, to show the effectiveness of CMAP. Of note, the incidences of transient and persistent PNI are lower in the studies that did use CMAP. Interestingly, the reported incidences of PNI in the studies that used CMAP were not due to failure of the CMAP approach. In the initial study conducted by Franceschi et al. [9], persistent PNI (0.7%) had occurred in 1 of 8 patients for whom the use of CMAP was not feasible. Furthermore, Mayazaki et al. [13] had used various CB positions in relation to the cardiac shadow under fluoroscopic guidance and concluded that deeper balloon positions (fluoroscopically more than one-third outside the cardiac shadow [13] were associated with a higher incidence of PNI, which has previously been described [32]. It can be speculated that the incidences of PNI might have been lower in both studies if CMAP were applicable in all patients and deeper balloon positions had been avoided, respectively. It is important to note that a direct comparison between the presented studies is limited due to the absence of differentiation between transient and persistent PNI, smaller sample sizes, the use of different CB sizes, different freezing methods, adoption of new ablation techniques midway through the study, and the use of different protective techniques (if any). In our study, we used a single, 180-s freeze technique with immediate balloon deflation as soon as a CMAP reduction less than 30% was observed, and used the proximal seal technique as initially described by Su et al. [11] to prevent PNI [13,26]. The exact position and depth of the inflated cryoballoon was assessed fluoroscopically in relation to the PV ostium with contrast medium before freezing via the endoluminal spiral mapping catheter (Achieve^™^, Medtronic).

**Table 5:** List of studies showing the incidence of transient and persistent PNI with and without CMAP.

Study	n	CB	Method	CMAP	Transient phrenic nerve injury (%)	Persistent phrenic nerve injury (%)
Martins, et al. 2014 [16]	81	CB-G2	Single 240 s freeze	No	24.7	0
Straube, et al. 2014 [17]	120	CB-G2	Single 240 s freeze	No	27.5	1.7
Fürnkranz, et al. 2014 [18]	55	CB-G2	240 s freeze + bonus freeze	No	7.2	5.4
Di Giovanni, et al. 2014 [19]	50	CB-G2	240 s freeze + bonus freeze	No	16	2
Chierchia et al., 2014 [23]	42	CB-G2	single 240 s freeze	No	19	7.1
Metzner, et al. 2014 [20]	50	CB-G2	Single 240 s freeze + bonus freeze	No	2.0	0
Ciconte, et al. 2015 [21]	143	CB-G2	Single 180 s freeze	No	6.3	3.5
Mugnai, et al. 2015 [22]	550	CB-G2	Single 240 s freeze + bonus freeze (80 patients), Single 180 s freeze (470 patients)	No	5.3	2
Prochnau, et al. 2018 [14]	88	CB-G2	Two 4-minute freezes + optional bonus freeze	No	N/A	2.4
Franceschi, et al. 2015 [7]	140	CB-G2	Single 180 s freeze + 180 s freeze if time to isolation under 60 s was not achieved	Yes	0	0.7
Meissner, et al. 2016 [2]	105	CB-G2	Two 240 s freezes	Yes	3.8	0
Miyazaki, et al. 2018 [13]	550	CB-G2	Single 180 s freeze	Yes	6.2	4

CB-G2 = second-generation cryoballoon (Arctic Front Advance (Medtronic, Inc., Minneapolis, MN, USA), CMAP = compound motor action potential, PNI = phrenic nerve injury, s = seconds

#### Other modalities to prevent PNI

Parikh et al. [27] had investigated additional monitoring modalities to prevent PNI. Other methods were also analysed, such as intracardiac echocardiography, auditory cardiotopography, and changes in femoral venous pressure. These techniques appeared most useful in confirming that PNI had already taken place, given they only began to manifest changes far into the pathophysiological process of phrenic nerve damage [28]. Therefore, the electromyographic approach was not only non-invasive and cost-efficient, but was the only method that showed a predictive value in the prevention of PNI [27].

#### Potential alternatives

Another strategy for the prevention of PNI could be the reduction of total freezing time. According to a recent study by Molenaar et al., a ‘tailored’ approach to PVI has been proposed [29]: application times less than 2 minutes affected the success of the left PV but not the right PV and resulted in less PNI [29]. In our study, we used a freezing time of 180 s in all PVs if real-time PVI occurred within 60 s. Further studies are required to assess whether reduced freezing times during cryoablation can further decrease the incidence of PNI.

One of the challenges with CMAP is the differentiation between a true CMAP reduction and a CMAP reduction caused by breathing excursions. Additional and available measuring tools could be considered to solve this issue. For example, an acceleromyograph, widely used in the field of anaesthesiology to reduce the risk of residual paralysis in the postoperative period, can be considered to quantify the weakness of diaphragmatic muscle excursions during ablation of the right PV. This approach could be considered in addition to CMAP, instead of relying on qualitative measures. To the best of our knowledge, this method has yet to be analyzed in patients undergoing PVI.

## Conclusion

Although the use of CMAP monitoring during CB-PVI of AF is easily applicable and reliable, It is important to note that the low incidence of PNI can not be solely attributed to the use of CMAP alone. It is the use of multiple factors, most importantly, using the proximal seal technique and avoidance of a deeper balloon position which in combination with CMAP reduces the number of PNI. All cases of PNI in this study were preceded by a reduction in CMAP amplitude, however, whether this finding might be challenged in the future by rare cases with specific patient characteristics is not known.
